# Supplementary material for: Computational structural studies of SGLT2-related polypharmacy
Source: PLoS One. 2025 Oct 17;20(10):e0334656. doi: 10.1371/journal.pone.0334656 (PMC12533862; doi:10.1371/journal.pone.0334656)
Supplement: Attachment — Submitted filename: Response.docx [file pone.0334656.s002.docx]

Dear Ahmed Elkamhawy,

We are very grateful to you and both reviewers for reviewing our submission and for the considered feedback we have received as a result. A point-by-point reply to the comments can be found below.

Journal requirements

1. The resubmitted version of this report conforms to the PLOS file style and naming guidelines. Thank you for bringing this to our attention.
2. The modelling, docking and docking analysis tools used for this study can be found at https://proteins.swan.ac.uk/modelling-portal/. This website hosts automated structural modelling and docking services based on validated published methods freely available for academic use and credited within the manuscript. An institutional email address is required to sign up and a report detailing the tools found on this website is in press. The code used for MD simulations is freely available through github at <https://github.com/bioinfkaustin/gromacs-on-colab>.
3. We are sorry that the supplementary data was not available. This has now been corrected, and the files have been appropriately released for public viewing on figshare. The data files can be found at <https://doi.org/10.6084/m9.figshare.28363397.v1>.

Reviewer 1

We would like to thank Dr. Shovonlal Gayen for their time and for the constructive suggestions. Our responses to their comments are below.

1. We have furnished the introduction with more background information of the protein, polypharmacy as a concept and the widespread use of SGLT2 inhibitors.
2. Significant additions have been made to the results section aiming to address this concern.
3. A discussion of the protein-ligand interactions in greater detail has been included in the revised results section. We hope this addresses both this comment and comment 2 above. New figures have also been included to describe the protein structure and the ligand interactions of the 6 compounds with the lowest median RMSD in the MD studies
4. We acknowledge that through reporting only 10 nanoseconds of data, the molecular dynamics (MD) elements of this study include relatively short simulations. This important aspect of study design was not a choice taken lightly. MD studies are a valuable addition to the assessment of docking results and have been validated for this purpose^1^. A validation study using the DUD-E dataset identified a root mean square deviation (RMSD) threshold of 5.5Å was robust in discerning true active poses in MD simulations of 10, 50 and 100 nanosecond durations. In this study the difference in sensitivity measured by the area under a ROC curve (AUC) between each simulation length was relatively small. The calculated AUC values for experiments using the DUD-E dataset were 0.806, 0.840 and 0.836 for simulations of 10, 50 and 100 nanoseconds respectively^1^. The current study is the second performed using a new study protocol for high-throughput docking-MD screening that aims to make such structural bioinformatics studies achievable and accessible to new researchers to the field and researchers in training. The first, a repurposing screen of AQP1 was published in PLOS One earlier this year^2^. This second study of SGLT2 has a significant methodological upgrade to the AQP1 study as each ligand was simulated three times with the median RMSD value used to determine whether a ligand remained bound. There are over 50 publications within the NCBI PubMed database^3–54^, in addition to our recent report of the aquaporin 1 virtual screen^2^, which have used MD simulations of 10 nanoseconds to draw conclusions.
   Despite a thorough literature review, we have been unable to identify any studies validating the use of RMSF, MMPBSA, and RG in short post-docking MD studies. Without this validation it is unclear whether these additional measurements would meaningfully enhance the study conclusions. Increasing the complexity of data analysis is contrary to the premise of the protocol under development which aims to broaden access to the field of structural bioinformatics and make studies feel more achievable to new researchers to the field.
   In conclusion, we assert that three simulations of 10 nanosecond duration with assessment of ligand binding based on median RMSD are provide a validated and reproducible method to judge docked ligand conformation stability. A concise explanation of this in contained within the methods of the revised manuscript.
5. Dapagliflozin, canagliflozin, empagliflozin and ertugliflozin are licensed medicinal compounds known to act against SGLT2. These compounds have been included within all steps of the analysis through design as positive control ligands. The results for these compounds suggest stable binding with the protein. We are unclear which other “standard inhibitors” of SGLT2 reviewer 1 would like included. We cannot think of any more appropriate known inhibitors that these 4 drugs in current clinical use. We would contest that there is a large body of evidence that these compounds act against SGLT2 including experimental crystal structures confirming the interaction^55–57^.

We note the concern raised in the set questions regarding statistical analysis and would like clarification on whether this relates to points 3, 4, and 5, to which we have provided a robust response, or another aspect of the study.

We were disappointed to learn that this reviewer did not feel the standard of English language within the submitted manuscript met expectations. Unfortunately, no comment regarding any specific language concern or typographical errors has been made to direct improvements to the manuscript or address any perceived deficits. We have therefore concentrated on making strenuous efforts to proofread the manuscript to remove typographical errors from the revised manuscript.

Reviewer 2

We would like to thank Reviewer 2 for their time and for the constructive suggestions. Our responses to their comments are below.

1. We wholeheartedly agree that *in vitro* validation of these in-silico findings would be extremely valuable. We are unfortunately not able to complete these studies. Our hope in presenting this manuscript is that a laboratory with the expertise and resources to perform *in vitro* studies of SGLT2 would be inspired to validate these results. We have reinforced our firm belief that these findings need *in vitro* or clinical validation in the revised manuscript.
2. We have not studied SGLT1, mostly because despite the sequence similarity this protein is not acknowledged as a target for existing SGLT2 selective inhibitors^55^. However, we agree that a similar study of SGLT1 would be an interesting avenue for further research and now mention this within the discussion section of the revised manuscript.
3. The problem with the supplementary data has been addressed. Thank you for bringing this to our attention. Please see comments to the editor. The data can be found at <https://doi.org/10.6084/m9.figshare.28363397.v1>.

References

1.	Guterres, H. & Im, W. Improving Protein-Ligand Docking Results with High-Throughput Molecular Dynamics Simulations. *J. Chem. Inf. Model.* **60**, 2189–2198 (2020).

2.	Lloyd, A. R., Austin-Muttitt, K. & Mullins, J. G. L. In silico drug repurposing at the cytoplasmic surface of human aquaporin 1. *PLoS ONE* **20**, e0314151 (2025).

3.	Agarwal, S. *et al.* An integrated computational approach of molecular dynamics simulations, receptor binding studies and pharmacophore mapping analysis in search of potent inhibitors against tuberculosis. *J Mol Graph Model* **83**, 17–32 (2018).

4.	Ali, M. A., Nath, A., Jannat, M. & Islam, M. M. Direct Synthesis of Diamides from Dicarboxylic Acids with Amines Using Nb2O5 as a Lewis Acid Catalyst and Molecular Docking Studies as Anticancer Agents. *ACS Omega* **6**, 25002–25009 (2021).

5.	Aliko, V. *et al.* ‘From shadows to shores’-quantitative analysis of CuO nanoparticle-induced apoptosis and DNA damage in fish erythrocytes: A multimodal approach combining experimental, image-based quantification, docking and molecular dynamics. *Sci Total Environ* **906**, 167698 (2024).

6.	Aloui, M. *et al.* QSAR modelling, molecular docking, molecular dynamic and ADMET prediction of pyrrolopyrimidine derivatives as novel Bruton’s tyrosine kinase (BTK) inhibitors. *Saudi Pharm J* **32**, 101911 (2024).

7.	Amini, R., Moradi, S., Najafi, R., Mazdeh, M. & Taherkhani, A. BACE1 Inhibition Utilizing Organic Compounds Holds Promise as a Potential Treatment for Alzheimer’s and Parkinson’s Diseases. *Oxid Med Cell Longev* **2024**, 6654606 (2024).

8.	Askari, F. S. *et al.* Digging for the discovery of SARS-CoV-2 nsp12 inhibitors: a pharmacophore-based and molecular dynamics simulation study. *Future Virol* (2022) doi:10.2217/fvl-2022-0054.

9.	Azam, M. A. & Jupudi, S. Insight into the structural requirements of thiophene-3-carbonitriles-based MurF inhibitors by 3D-QSAR, molecular docking and molecular dynamics study. *J Recept Signal Transduct Res* **37**, 522–534 (2017).

10.	Azam, M. A. & Thathan, J. Pharmacophore generation, atom-based 3D-QSAR and molecular dynamics simulation analyses of pyridine-3-carboxamide-6-yl-urea analogues as potential gyrase B inhibitors. *SAR QSAR Environ Res* **28**, 275–296 (2017).

11.	Bandaru, S. *et al.* Molecular dynamic simulations reveal suboptimal binding of salbutamol in T164I variant of β2 adrenergic receptor. *PLoS One* **12**, e0186666 (2017).

12.	Chaudhuri, A., Bera, A. K., Sarkar, I. & Chakraborty, S. Insights from Analysis of Binding Sites of Human Meprins: Screening of Inhibitors by Molecular Dynamics Simulation Study. *Comb Chem High Throughput Screen* **19**, 246–258 (2016).

13.	Chauhan, D. *et al.* Design, synthesis, biological evaluation, and molecular modeling studies of rhodanine derivatives as pancreatic lipase inhibitors. *Arch Pharm (Weinheim)* **352**, e1900029 (2019).

14.	Chen, P.-Y. & Han, L.-T. Study on the molecular mechanism of anti-liver cancer effect of Evodiae fructus by network pharmacology and QSAR model. *Front Chem* **10**, 1060500 (2022).

15.	da Silva, G. D. *et al.* In vitro and in silico studies of the larvicidal and anticholinesterase activities of berberine and piperine alkaloids on Rhipicephalus microplus. *Ticks Tick Borne Dis* **12**, 101643 (2021).

16.	Das, B. K., Pv, P. & Chakraborty, D. Computational insights into factor affecting the potency of diaryl sulfone analogs as Escherichia coli dihydropteroate synthase inhibitors. *Comput Biol Chem* **78**, 37–52 (2019).

17.	Dhameliya, T. M., Nagar, P. R. & Gajjar, N. D. Systematic virtual screening in search of SARS CoV-2 inhibitors against spike glycoprotein: pharmacophore screening, molecular docking, ADMET analysis and MD simulations. *Mol Divers* **26**, 2775–2792 (2022).

18.	Gahtori, J., Pant, S. & Srivastava, H. K. Modeling antimalarial and antihuman African trypanosomiasis compounds: a ligand- and structure-based approaches. *Mol Divers* **24**, 1107–1124 (2020).

19.	Guerrero-Perilla, C., Bernal, F. A. & Coy-Barrera, E. Insights into the interaction and binding mode of a set of antifungal azoles as inhibitors of potential fungal enzyme-based targets. *Mol Divers* **22**, 929–942 (2018).

20.	Halder, S. K. & Elma, F. In silico identification of novel chemical compounds with antituberculosis activity for the inhibition of InhA and EthR proteins from Mycobacterium tuberculosis. *J Clin Tuberc Other Mycobact Dis* **24**, 100246 (2021).

21.	Huang, X., Dorhout Mees, E., Vos, P., Hamza, S. & Braam, B. Everything we always wanted to know about furosemide but were afraid to ask. *Am J Physiol Renal Physiol* **310**, F958-971 (2016).

22.	Jamal, S., Grover, A. & Grover, S. Machine Learning From Molecular Dynamics Trajectories to Predict Caspase-8 Inhibitors Against Alzheimer’s Disease. *Front Pharmacol* **10**, 780 (2019).

23.	Johari, S., Sharma, A., Sinha, S. & Das, A. Integrating pharmacophore mapping, virtual screening, density functional theory, molecular simulation towards the discovery of novel apolipoprotein (apoE ε4) inhibitors. *Comput Biol Chem* **79**, 83–90 (2019).

24.	Kamel, E. M. *et al.* Molecular modeling and DFT studies on the antioxidant activity of Centaurea scoparia flavonoids and molecular dynamics simulation of their interaction with β-lactoglobulin. *RSC Adv* **13**, 12361–12374 (2023).

25.	Kamel, E. M. *et al.* Xanthine Oxidase Inhibitory Activity of Euphorbia peplus L. Phenolics. *Comb Chem High Throughput Screen* **25**, 1336–1344 (2022).

26.	Kausar, M. A. *et al.* Identifying the alpha-glucosidase inhibitory potential of dietary phytochemicals against diabetes mellitus type 2 via molecular interactions and dynamics simulation. *Cell Mol Biol (Noisy-le-grand)* **67**, 16–26 (2022).

27.	Khan, A. A., Baildya, N., Dutta, T. & Ghosh, N. N. Inhibitory efficiency of potential drugs against SARS-CoV-2 by blocking human angiotensin converting enzyme-2: Virtual screening and molecular dynamics study. *Microb Pathog* **152**, 104762 (2021).

28.	Khan, M. K. A., Akhtar, S. & Arif, J. M. Development of In Silico Protocols to Predict Structural Insights into the Metabolic Activation Pathways of Xenobiotics. *Interdiscip Sci* **10**, 329–345 (2018).

29.	Khan, M. F. *et al.* Dibenzepinones, dibenzoxepines and benzosuberones based p38α MAP kinase inhibitors: Their pharmacophore modelling, 3D-QSAR and docking studies. *Comput Biol Med* **110**, 175–185 (2019).

30.	Khedr, M. A., Mohafez, O. M. M. & Al-Haider, I. A. Virtual Screening-Based Discovery of Potent Hypoglycemic Agents: In Silico, Chemical Synthesis and Biological Study. *Curr Comput Aided Drug Des* **16**, 741–756 (2020).

31.	Makeneni, S., Thieker, D. F. & Woods, R. J. Applying Pose Clustering and MD Simulations To Eliminate False Positives in Molecular Docking. *J. Chem. Inf. Model.* **58**, 605–614 (2018).

32.	Malekipour, M. H., Shirani, F., Moradi, S. & Taherkhani, A. Cinnamic acid derivatives as potential matrix metalloproteinase-9 inhibitors: molecular docking and dynamics simulations. *Genomics Inform* **21**, e9 (2023).

33.	Masumi, M. *et al.* Methicillin-Resistant Staphylococcus aureus: Docking-Based Virtual Screening and Molecular Dynamics Simulations to Identify Potential Penicillin-Binding Protein 2a Inhibitors from Natural Flavonoids. *Int J Microbiol* **2022**, 9130700 (2022).

34.	Modi, P., Patel, S. & Chhabria, M. Structure-based design, synthesis and biological evaluation of a newer series of pyrazolo[1,5-a]pyrimidine analogues as potential anti-tubercular agents. *Bioorg Chem* **87**, 240–251 (2019).

35.	Moussa, N., Hassan, A. & Gharaghani, S. Pharmacophore model, docking, QSAR, and molecular dynamics simulation studies of substituted cyclic imides and herbal medicines as COX-2 inhibitors. *Heliyon* **7**, e06605 (2021).

36.	Muniz Seif, E. J., Icimoto, M. Y. & Silva Júnior, P. I. In silico bioprospecting of receptors associated with the mechanism of action of Rondonin, an antifungal peptide from spider Acanthoscurria rondoniae haemolymph. *In Silico Pharmacol* **12**, 55 (2024).

37.	Nagar, P. R., Gajjar, N. D. & Dhameliya, T. M. In search of SARS CoV-2 replication inhibitors: Virtual screening, molecular dynamics simulations and ADMET analysis. *J Mol Struct* **1246**, 131190 (2021).

38.	Omoboyowa, D. A. *et al.* Structure-based discovery of selective CYP17A1 inhibitors for Castration-resistant prostate cancer treatment. *Biol Methods Protoc* **7**, bpab026 (2022).

39.	Omoboyowa, D. A., Balogun, T. A., Omomule, O. M. & Saibu, O. A. Identification of Terpenoids From Abrus precatorius Against Parkinson’s Disease Proteins Using In Silico Approach. *Bioinform Biol Insights* **15**, 11779322211050757 (2021).

40.	Parcha, P. *et al.* Identification of natural inhibitors of Bcr-Abl for the treatment of chronic myeloid leukemia. *Chem Biol Drug Des* **90**, 596–608 (2017).

41.	Patel, H. M., Shaikh, M., Ahmad, I., Lokwani, D. & Surana, S. J. BREED based de novo hybridization approach: generating novel T790M/C797S-EGFR tyrosine kinase inhibitors to overcome the problem of mutation and resistance in non small cell lung cancer (NSCLC). *J Biomol Struct Dyn* **39**, 2838–2856 (2021).

42.	Patel, H. M., Ahmad, I., Pawara, R., Shaikh, M. & Surana, S. In silico search of triple mutant T790M/C797S allosteric inhibitors to conquer acquired resistance problem in non-small cell lung cancer (NSCLC): a combined approach of structure-based virtual screening and molecular dynamics simulation. *J Biomol Struct Dyn* **39**, 1491–1505 (2021).

43.	Pola, M., Rajulapati, S. B., Potla Durthi, C., Erva, R. R. & Bhatia, M. In silico modelling and molecular dynamics simulation studies on L-Asparaginase isolated from bacterial endophyte of Ocimum tenuiflorum. *Enzyme Microb Technol* **117**, 32–40 (2018).

44.	Rustamzadeh, A. *et al.* Targeting Caspases 3/6 and Cathepsins L/B May Decrease Laminopathy-Induced Apoptosis in Alzheimer’s Disease. *J Alzheimers Dis* **101**, 211–221 (2024).

45.	Shakibay Senobari, Z. *et al.* Chromone-embedded peptidomimetics and furopyrimidines as highly potent SARS-CoV-2 infection inhibitors: docking and MD simulation study. *BMC Res Notes* **16**, 224 (2023).

46.	Shrivastava, A. K., Kumar, S., Sahu, P. S. & Mahapatra, R. K. In silico identification and validation of a novel hypothetical protein in Cryptosporidium hominis and virtual screening of inhibitors as therapeutics. *Parasitol Res* **116**, 1533–1544 (2017).

47.	Sridhar, S. N. C., Palawat, S. & Paul, A. T. Design, synthesis, evaluation, and molecular modeling studies of indolyl oxoacetamides as potential pancreatic lipase inhibitors. *Arch Pharm (Weinheim)* **353**, e2000048 (2020).

48.	Suman, null, Chaudhary, M. & Nain, V. In silico identification and evaluation of Bacillus subtilis cold shock protein B (cspB)-like plant RNA chaperones. *J Biomol Struct Dyn* **39**, 841–850 (2021).

49.	Tania, M. *et al.* Cordycepin Downregulates Cdk-2 to Interfere with Cell Cycle and Increases Apoptosis by Generating ROS in Cervical Cancer Cells: in vitro and in silico Study. *Curr Cancer Drug Targets* **19**, 152–159 (2019).

50.	Tripuraneni, N. S. & Azam, M. A. A combination of pharmacophore modeling, atom-based 3D-QSAR, molecular docking and molecular dynamics simulation studies on PDE4 enzyme inhibitors. *J Biomol Struct Dyn* **34**, 2481–2492 (2016).

51.	Uba, A. I. & Yelekçi, K. Carboxylic acid derivatives display potential selectivity for human histone deacetylase 6: Structure-based virtual screening, molecular docking and dynamics simulation studies. *Comput Biol Chem* **75**, 131–142 (2018).

52.	Verma, A. K. *et al.* Identification of 1, 2, 4-Triazine and Its Derivatives Against Lanosterol 14-Demethylase (CYP51) Property of Candida albicans: Influence on the Development of New Antifungal Therapeutic Strategies. *Front Med Technol* **4**, 845322 (2022).

53.	Wan, Y. *et al.* In silico studies of diarylpyridine derivatives as novel HIV-1 NNRTIs using docking-based 3D-QSAR, molecular dynamics, and pharmacophore modeling approaches. *RSC Adv* **8**, 40529–40543 (2018).

54.	Zhao, X. *et al.* Synthesis and biological evaluation of 6-hydroxyl C-aryl glucoside derivatives as novel sodium glucose co-transporter 2 (SGLT2) inhibitors. *Bioorg Med Chem Lett* **28**, 2201–2205 (2018).

55.	Ferrannini, E. Sodium-Glucose Co-transporters and Their Inhibition: Clinical Physiology. *Cell Metab* **26**, 27–38 (2017).

56.	Mudaliar, S., Polidori, D., Zambrowicz, B. & Henry, R. R. Sodium-Glucose Cotransporter Inhibitors: Effects on Renal and Intestinal Glucose Transport: From Bench to Bedside. *Diabetes Care* **38**, 2344–2353 (2015).

57.	Niu, Y. *et al.* Structural basis of inhibition of the human SGLT2–MAP17 glucose transporter. *Nature* **601**, 280–284 (2022).
